# A bibliometric and visualization study of global research trends in sacral Tarlov cyst from 2000 to 2022

**DOI:** 10.3389/fsurg.2023.1301739

**Published:** 2024-01-03

**Authors:** Yang Lu, Luyao Bao, Nan Wang, Shuang Chen, Yuzhang Qian, Jun Gu, Ran Kang, Lin Xie

**Affiliations:** ^1^ Department of Orthopaedics, Affiliated Hospital of Integrated Traditional Chinese and Western Medicine, Nanjing University of Chinese Medicine, Nanjing, China; ^2^ Department of Traditional Chinese Medicine, The First Affiliated Hospital with Nanjing Medical University, Nanjing, China

**Keywords:** bibliometrics, visualization study, sacral Tarlov cyst, research trends, hot spots analysis

## Abstract

**Background:**

Symptomatic sacral Tarlov cyst (STC) exerts a significant negative impact on the patient's quality of life, highlighting the significance of the increasing number of studies on STC. However, bibliometric analyses in this research field are scarce. Thus, this study aims to provide a comprehensive knowledge structure and identify the research trends of STC through bibliometrics.

**Methods:**

Articles related to STC from 2000 to 2022 were sourced from the Web of Science Core Collection database. VOSviewer 1.6.16, CiteSpace 6.1.6, GraphPad Prism 8.2.1 and R-package “bibliometrix” were used to analyse the data and generate knowledge maps.

**Results:**

A total of 930 studies on STC from 2000 to 2022 were included. The findings revealed a consistent yet upward trend in the number of annual publications in this field. The United States, China and Turkey were the most prolific and influential countries contributing to this field, with the University of Illinois, the University of Maryland and the National Institute of Standards & Technology being the most notable research institutions. Key journals include *World Neurosurgery* [Impact Factor (IF) = 2.210], *Journal of Vascular Surgery* (IF = 4.860) and *Journal of Neurosurgery-Spine* (IF = 3.467). Additionally, Tarlov Mj, Tarlov E and Zachariah Mr exhibit the highest number of publications, making them the leading authors in this field. A twenty-year retrospection of research trends using keyword analysis reveals four principal directions, namely “definition”, “pathogenesis”, “diagnosis” and “treatment”. Currently, therapeutic surgical intervention is the key treatment for this disease, with future treatments primarily hinging on minimally invasive methodologies rooted in microendoscopic and endoscopic techniques.

**Conclusion:**

This pioneering, comprehensive scientific bibliometric study provides a holistic summary of STC research trends and hot spots spanning the past 22 years. The results identify existing research frontiers and chart maps for future studies, serving as a valuable reference for scholars vested in this field.

## 1. Introduction

Spinal disorders involve structural changes in the components of the spine, including the bones, intervertebral discs, muscles, and ligaments, which may stimulate the spinal cord and spinal nerves (1). Neurological disorders encompass a wide range of conditions, including cerebrovascular diseases, neuroimmune disorders, central nervous system infections, peripheral nerve diseases, and spinal cord diseases (2, 3). With the continuous improvement of imaging technology and the growing body of literature, some rare spinal disorders and neurological conditions are gradually gaining recognition. Among them, Sacral Tarlov cyst (STC), initially reported by Tarlov in 1938, refers to cysts within the sacral nerve tract membrane (4). Its prevalence ranges between 1.5% and 4.6% (5–7), with a higher incidence observed among women. Although most patients with STC remain asymptomatic, approximately 20%–25% of patients with STC experience symptoms such as pain, numbness, fatigue, urinary and faecal function dysfunction and other symptoms (8). With the further development of CT, MRI and endoscopic technology, STC has gradually garnered attention. Presently, the pathogenic factors of STC remain unclear; however, congenital developmental abnormalities, secondary trauma and inflammations are speculated to be contributors (9–11). In terms of pathogenesis, the “ball-valve” mechanism garners expert endorsement (12, 13), yet its substantiation remains controversial. Current treatment options for STC include conservative or surgical treatment modalities (14–16). A patient with symptomatic STC can undergo conservative treatment management involving medication or physical therapy. Notably, surgical intervention has been demonstrated to be a viable option for cysts exceeding a diameter of 1.5 cm accompanied by nerve root symptoms, yielding improved clinical outcomes (17). In the past decades, researchers have explored various surgical treatment options for STC; however, several facets, such as the aetiology and pathogenesis of STC, clinical image prediction, advanced treatment technologies and research orientations, remain controversial (9).

Bibliometric analysis has emerged as an invaluable scientific tool for the quantitative analysis of scholarly literature within a particular knowledge field using various statistical and linguistic methods (18–21). this technique plays a pivotal role in elucidating knowledge structures by processing bibliographic elements, such as journals, authors and institutions (22). Bibliometrics are instrumental in clarifying research trends and historical trajectories of a certain disease (23). Despite its widespread use, a quantitative analysis of STC studies is yet to be undertaken. Thus, we analysed STC studies to evaluate major research clusters and popular research orientations, thereby predicting future research trends or hot spots in this field. Furthermore, it also aids in the exploration of the epidemiology, pathogenesis, treatment methods and prognosis of STC.

## 2. Materials and methods

### 2.1. Search strategy and data sources

**Figure 1:**
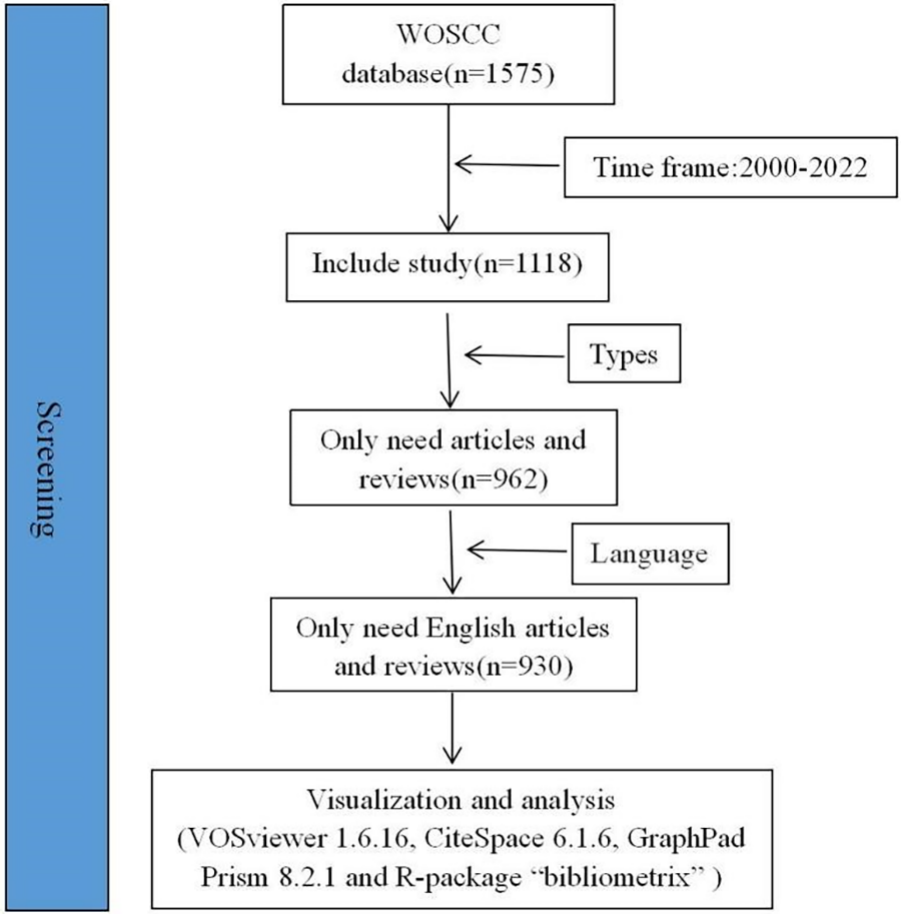
Flow chart of the selection process.

The data for this study was sourced from the Web of Science Core Collection (WOSCC), which is deemed the most suitable database for bibliometric analysis due to its credible information and rigorous evaluation process (21, 24, 25). All data collection was performed on 1st March 2023, encompassing literature published from 1st January 2000 to 31st December 2022. The search terms selected included “Sacral Tarlov Cyst” OR “Sacral Tarlov Cysts” OR “Tarlov Cyst” OR “Tarlov Cysts” OR “Sacral Cyst” OR “Sacral Cysts” OR “Sacral Tarlov” OR “Tarlov” OR “Sacral Perineural Cyst” OR “Sacral Perineural Cysts”. Articles and reviews were exclusively considered, with the language restricted to English. A total of 930 articles were included in this research, and the exported literatures were stored as full records, while references were formatted and stored as plain text files. The search strategy is summarised in Figure 1.

### 2.2. Bibliometric analysis

### 2.3. Annual publication performance

**Figure 2:**
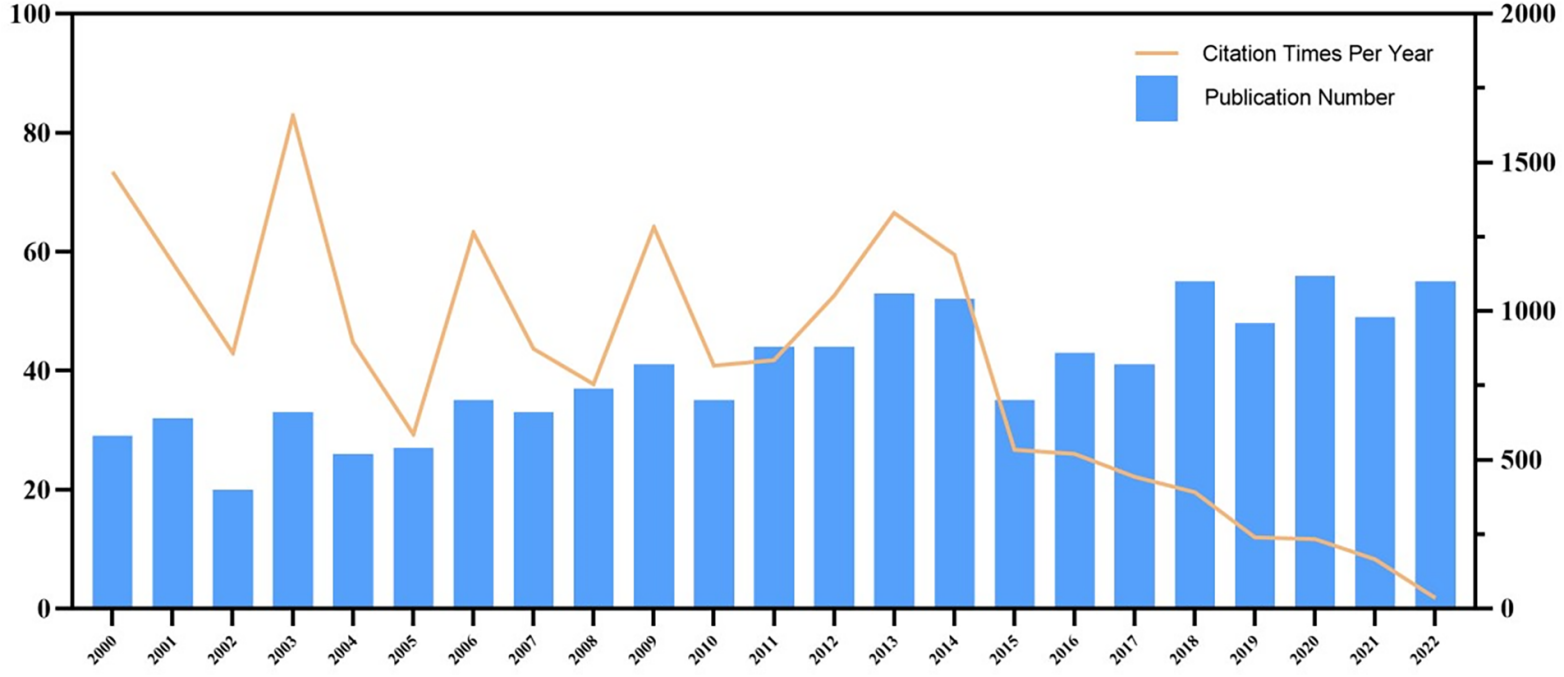
Number of publications and citations from 2000 to 2022.

The number of published literature in each period reﬂects the research trend in this ﬁeld. A total of 930 publications were found from 2000 to 2022. As shown in Figure 2, although the number of publications fluctuated from year to year, it showed a general upward trend. The lowest number of publications was recorded in 2003 (*n* = 19), whereas the number increased between 2018 and 2022. In 2020, 58 pieces of literature were published, recording the highest number so far. This peak is indicative of the increasing number of studies on STC, which has become a research hot spot. Before 2013, the annual citation rates exhibited a fluctuating trend; however, since 2013, although the number of articles has been increasing, the annual citation rates exhibit a significant downward trend. This trend suggests the potential onset of a bottleneck phase within the research field or the emergence of theoretical difficulties or technical complexities. It is imperative for future research to emphasise integrity and innovation.

### 2.4. Countries and regions analysis

The top 10 most productive countries are presented in Table 1. Although many countries have contributed to the field of STC research, only a few counties contribute majorly to the publication number. The top three countries published a cumulative of 568 papers (61.1%). The United States (*n* = 313, 33.7%) recorded the highest publication, followed by China (*n* = 143, 15.4%), Turkey (*n* = 112, 12.0%), Japan (*n* = 71, 7.6%) and Korea (*n* = 41, 4.4%).

**Table 1:** The top 10 countries or regions in STC research.

Rank	Region	Frequency	Rank	Region	Total citations
1	United States	313	1	United States	9,830
2	China	143	2	China	2,087
3	Turkey	112	3	Turkey	1,504
4	Japan	71	4	Japan	933
5	Korea	41	5	Korea	661
6	India	28	6	France	423
7	Italy	20	7	Germany	335
8	United Kingdom	19	8	Italy	330
9	France	17	9	Switzerland	326
10	Germany	17	10	United Kingdom	316

**Figure 3:**
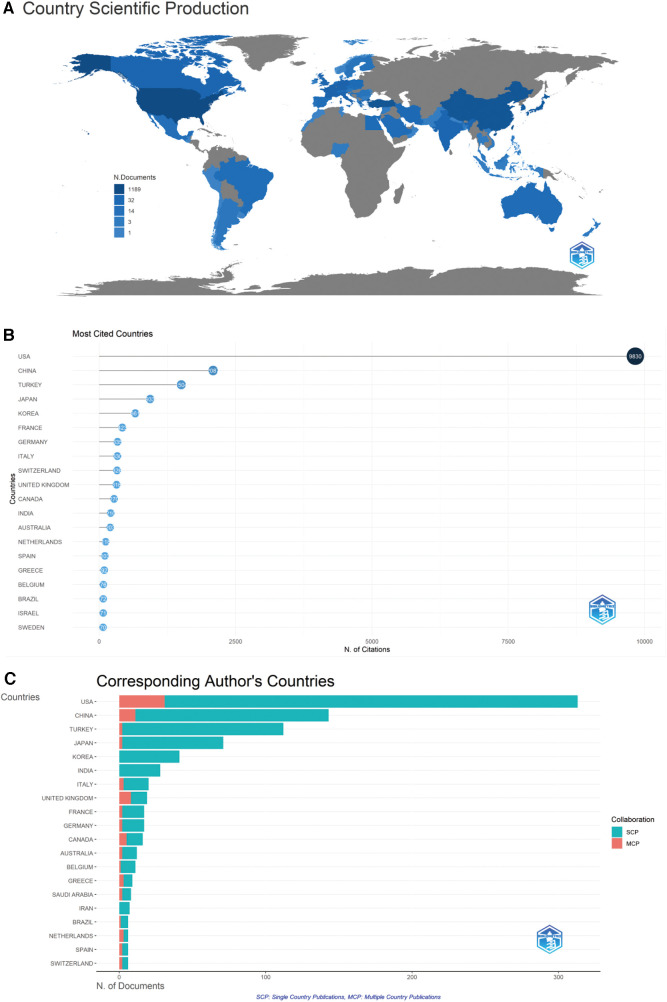
Global distribution of publications in STC research. (**A**) Geographic Map of Publication Source. (**B**) The top 20 most cited countries in the publication of STC research. (**C**) The top 20 corresponding author’s countries in the publication of STC research.

**Figure 4:**
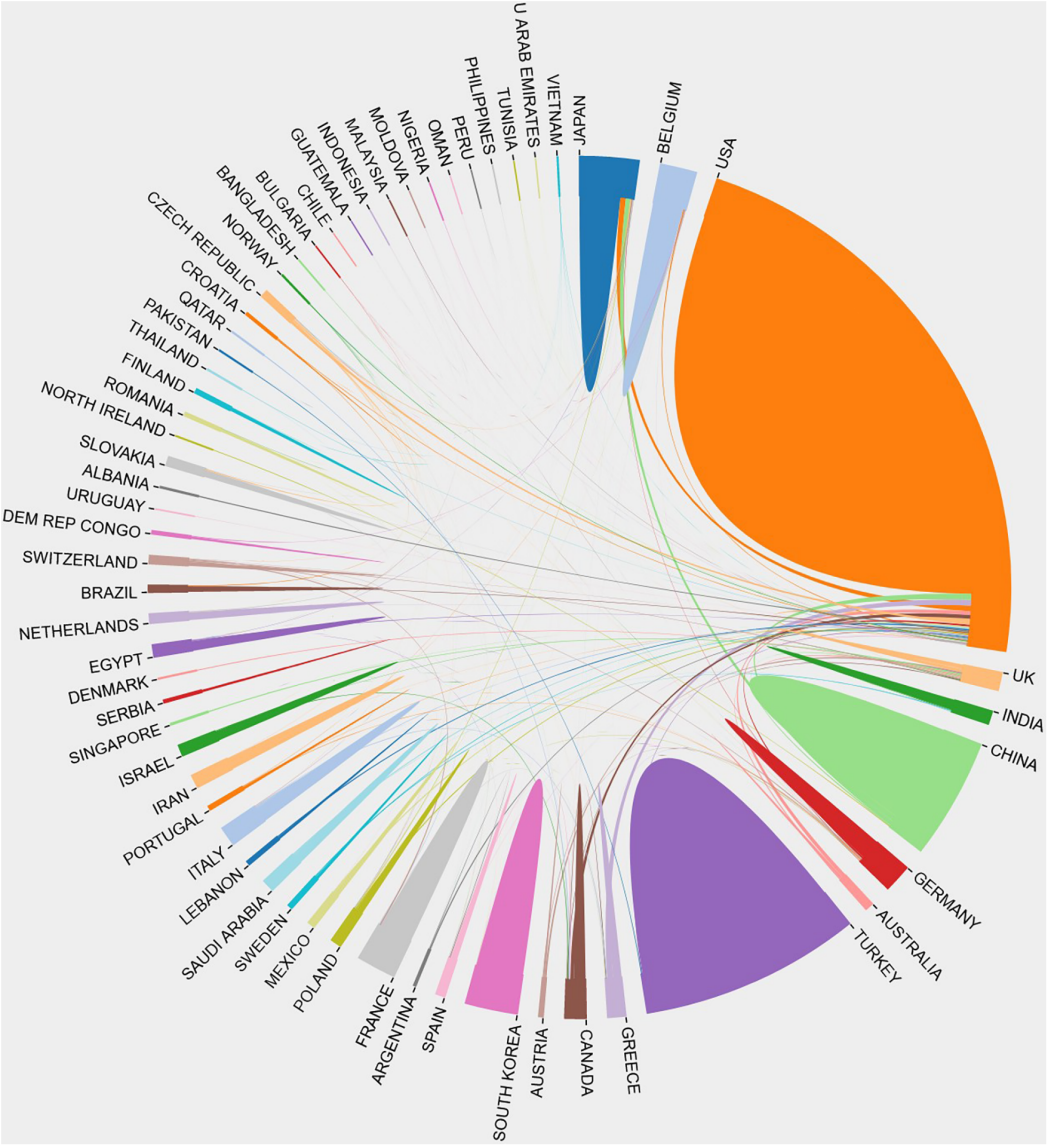
Chord chart of cooperation between countries.

R-package “bibliometrix” software facilitated the visualisation of these national differences (Figure 3A). As shown in the global map, the darker the colour, the greater the publication volume. Analysis of total citations (Table 1, Figure 3B) revealed that the United States had the most total citations (9,830), followed by China (total citations = 2,087), Turkey (total citations = 1,504), Japan (total citations = 933) and Korea (total citations = 661). Among the countries, USA ranks ﬁrst in terms of the number of corresponding authors, including single-country publications and multiple-country publications (Figure 3C). Inter-country cooperation patterns were graphically represented using an inter-country cooperation chord chart (Figure 4), showcasing the United States as a vital hub. While the USA and Japan maintained close ties with several countries, China and Turkey, despite their prolific publication number, displayed fewer collaborative ties, highlighting the need for enhanced international exchange in future endeavours.

### 2.5. Institutions analysis

**Figure 5:**
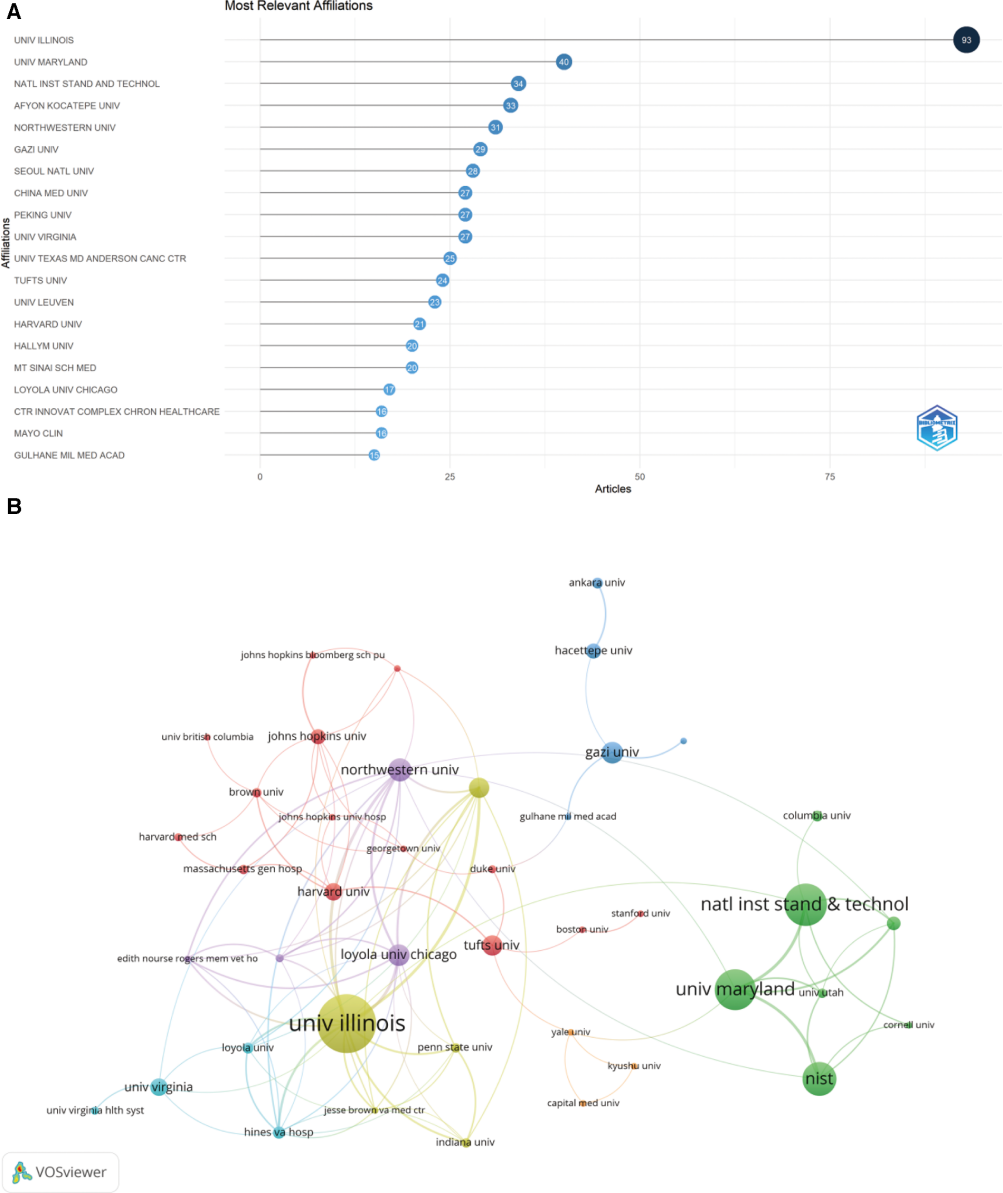
Number of publications and cooperation among institutions in STC research. (**A**) The top 20 institutions in the publication of STC research. (**B**) Cooperation network map of institutions in STC research. STC, Sacral Tarlov cyst.

Analysis of WOSCC yielded 67 institutions contributing to STC research, each publishing five or more articles. The top 20 most prolific institutions are presented in Table 2. The University of Illinois secured the leading position (USA, *n* = 93), followed by the University of Maryland (USA, *n* = 40), the National Institute of Standards & Technology (USA, *n* = 34) and Afyon Kocatepe University (Turkey, *n* = 33) (Figure 5A).

**Table 2:** The top 20 institutions in STC research.

Rank	Institution	Country	*n*
1	University of Illinois	United States	93
2	University of Maryland	United States	40
3	National Institute of Standards & Technology	United States	34
4	Afyon Kocatepe University	Turkey	33
5	Northwestern University	United States	31
6	Gazi University	Turkey	29
7	Seoul National University	South Korea	28
8	China Medical University	China	27
9	Peking University	China	27
10	University of Virginia	United States	27
11	The University of Texas MD Anderson Cancer Center	United States	25
12	Tufts University	United States	24
13	Catholic University of Leuven	Belgium	23
14	Harvard University	United States	21
15	Hallym University	South Korea	20
16	Mount Sinai Medical College	United States	20
17	Loyola University Chicago	United States	17
18	Center of Innovation for Complex Chronic Healthcare	United States	16
19	Mayo clinic	United States	16
20	Gülhane Military Medical Academy	Turkey	15

Subsequently, VOSviewer software was used to elucidate a collaborative network based on the number and connections of papers of each institution. As shown in Figure 5B, the cooperation between the University of Illinois, Loyola University Chicago, The University of Texas MD Anderson Cancer Center, Indiana University and Northwestern University was robust. Additionally, active cooperation was observed between Harvard University, Johns Hopkins University, Brown University and Georgetown University. Notably, despite their high publication counts, China Medical University and Peking University had fewer collaborations with other institutions.

### 2.6. Journal analysis

**Figure 6:**
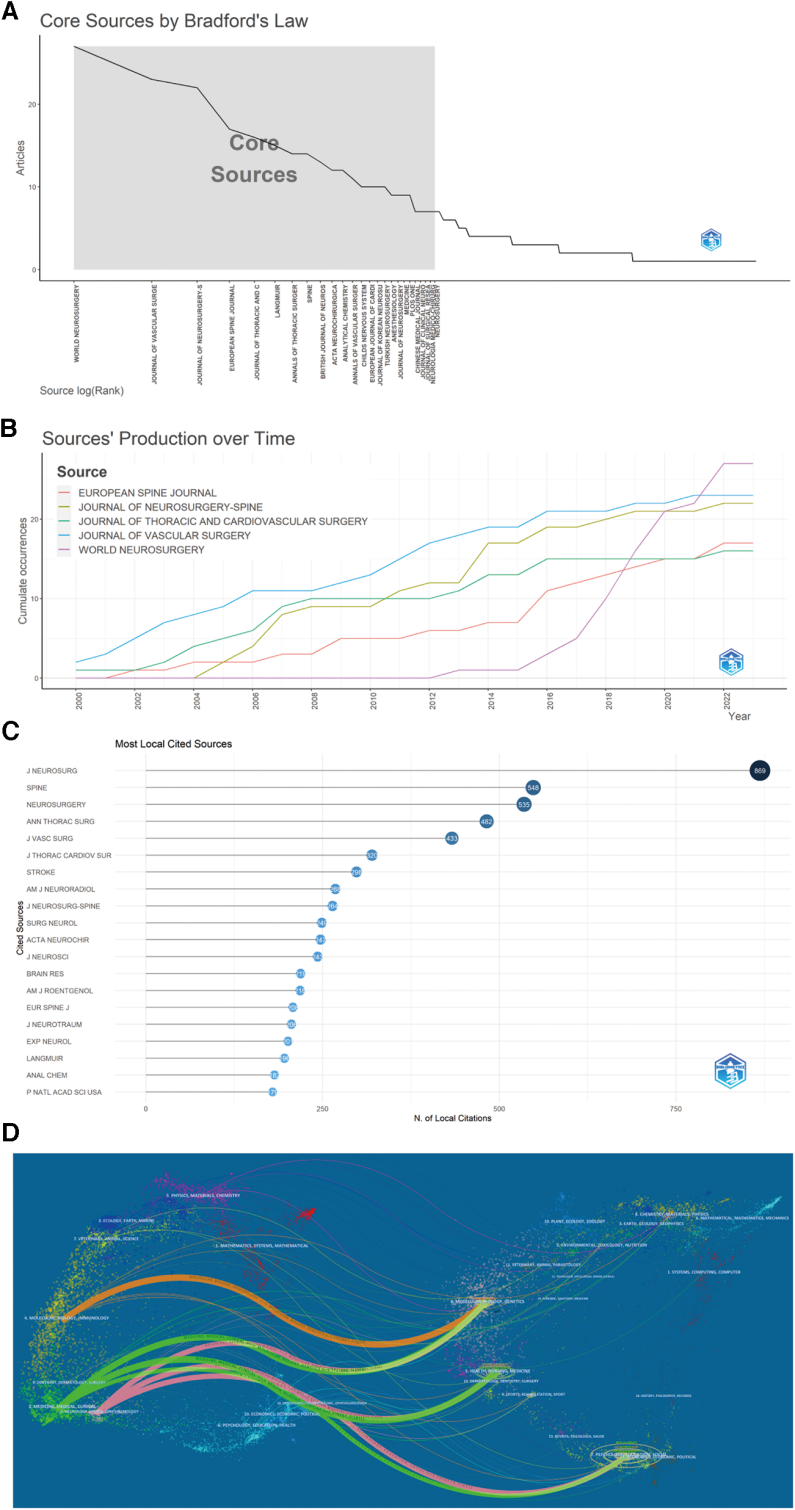
Journal analysis in STC research. (**A**) Core Sources by Bradford’s Law. (**B**) The top five Sources’ Production over time. (**C**) The top 20 most locally cited sources in STC research. (**D**) Dual map overlay of journals in STC research. STC, Sacral Tarlov cyst.

The journal publication volumes are summarized in Table 3. *World Neurosurgery* [*n* = 27, Impact Factor (IF) = 2.210], *Journal of Vascular Surgery* (*n* = 23, IF = 4.860) and *Journal of Neurosurgery-Spine* (*n* = 22, IF = 3.467) were identified as the three most proliﬁc journals in the field, followed by *European Spine Journal* (*n* = 17, IF = 2.721) and *Journal of Thoracic and Cardiovascular Surgery* (*n* = 16, IF = 6.439), Among the top 20 journals, *Anesthesiology* (IF = 9.198) exhibited the highest impact factor. This information is further represented in accordance with Bradford's law (Figure 6A). The R-package “bibliometrix” and VOSviewer were used to perform a visual analysis of these published literature. Examination of the annual publication volume of the top five journals revealed a consistent rise over time (Figure 6B).

**Table 3:** The top 20 journals in STC research.

Rank	Journal	*n*	Category^a^	IF(2021)^b^
1	*World Neurosurgery*	27	Surgery Clinical Neurology	2.210
2	*Journal Of Vascular Surgery*	23	Peripheral Vascular Disease Surgery	4.860
3	*Journal Of Neurosurgery-Spine*	22	Surgery Clinical Neurology	3.467
4	*European Spine Journal*	17	Orthopedics Clinical Neurology	2.721
5	*Journal Of Thoracic And Cardiovascular Surgery*	16	Surgery Cardiac & Cardiovascular Systems Respiratory System	6.439
6	*Langmuir*	15	Chemistry, Multidisciplinary Materials Science, Multidisciplinary Chemistry, Physical	4.331
7	*Annals Of Thoracic Surgery*	14	Surgery Cardiac & Cardiovascular Systems Respiratory System	5.113
8	*Spine*	14	Orthopedics Clinical Neurology	3.269
9	*British Journal Of Neurosurgery*	13	Surgery Clinical Neurology	1.124
10	*Acta Neurochirurgica*	12	Surgery Clinical Neurology	2.816
11	*Analytical Chemistry*	12	Chemistry, Analytical	8.008
12	*Annals Of Vascular Surgery*	11	Peripheral Vascular Disease Surgery	1.607
13	*Childs Nervous System*	10	Pediatrics Surgery Clinical Neurology	1.532
14	*European Journal Of Cardio-Thoracic Surgery*	10	Surgery Cardiac & Cardiovascular Systems Respiratory System	4.534
15	*Journal Of Korean Neurosurgical Society*	10	Surgery Clinical Neurology	2.249
16	*Turkish Neurosurgery*	10	Surgery Clinical Neurology	0.972
17	*Anesthesiology*	9	Anesthesiology	9.198
18	*Journal Of Neurosurgery*	9	Surgery Clinical Neurology	5.526
19	*Medicine*	9	Medicine, General & Internal	1.817
20	*Plos One*	9	Multidisciplinary Sciences	3.752

^a^ Category based on SCIE/SSCI.

^b^ IF from 2021 journal impact factor.

Regarding locally cited sources (Figure 6C), *The Journal of Neurosurgery* had the highest number of citations (*n* = 869, IF = 5.526), followed by *Spine* (*n* = 548, IF = 3.269). The dual map overlay of these journals, as facilitated by CiteSpace 6.1.6 software, illustrates the citing journals on the left side and the cited journals on the right. Seven primary citation relationships marked by three distinct colours were identified (Figure 6D). The orange colour (*z*-score = 3.11) revealed that the literature published in/molecular/biology/immunology journals were frequently cited in molecular/biology/genetics journals. The green path (*z*-score = 5.37) with the highest *z*-score revealed that the literature in the field of medicine/medical/clinical research mainly referred to the journals of/health/nursing/medicine. The pink path (*z*-score = 2.60) revealed that the works of literature published in neurology/sports/ophthalmology journals heavily cited from psychology/education/social journals.

### 2.7. Contributions of authors

In the field of STC research, the top five authors with the most publications were identified to be Tarlov Mj (*n* = 50), Tarlov E (*n* = 45), Zachariah Mr (*n* = 18), Kern Ja (*n* = 15) and Kron Il (*n* = 15). In the database, the top five authors published 15.4% of the total number. The top five co-cited authors with the most citations were Tarlov Mj (citations = 400), Paulsen Rd (citations = 115), Voyadzis Jm (citations = 106), Nabors Mw (citations = 98) and Coselli Js (citations = 91) (Table 4).

**Table 4:** The top 20 authors and Co-cited authors in STC.

Rank	Authors			Co-cited Authors	
	Name	Articles	Citations	Name	Citations
1	Tarlov Mj	50	133	Tarlov Mj	400
2	Tarlov E	45	87	Paulsen Rd	115
3	Zachariah Mr	18	88	Voyadzis Jm	106
4	Kern Ja	15	74	Nabors Mw	98
5	Kron Il	15	74	Coselli Js	91
6	Zenk Sn	15	16	Svensson Lg	86
7	Tribble Cg	14	73	Langdown Aj	77
8	Pease Lf	12	71	Mummaneni Pv	71
9	Stroupe Kt	12	14	Patel Mr	56
10	Guha S	11	34	Caspar W	55
11	Hynes Dm	11	12	Bartels Rhma	51
12	Linden J	11	57	Guo Ds	51
13	Zangmeister Ra	11	41	Pease Lf	51
14	Bruyninckx F	9	31	Lucantoni C	48
15	Colak A	9	20	Murphy K	48
16	Dankaerts W	9	31	Cassada Dc	47
17	Tsai Dh	9	64	Etz Cd	47
18	Wing C	9	14	Tanaka M	46
19	Hulens M	8	6	Park Hj	45
20	Jiang Xj	8	33	Sakurai M	44

**Figure 7:**
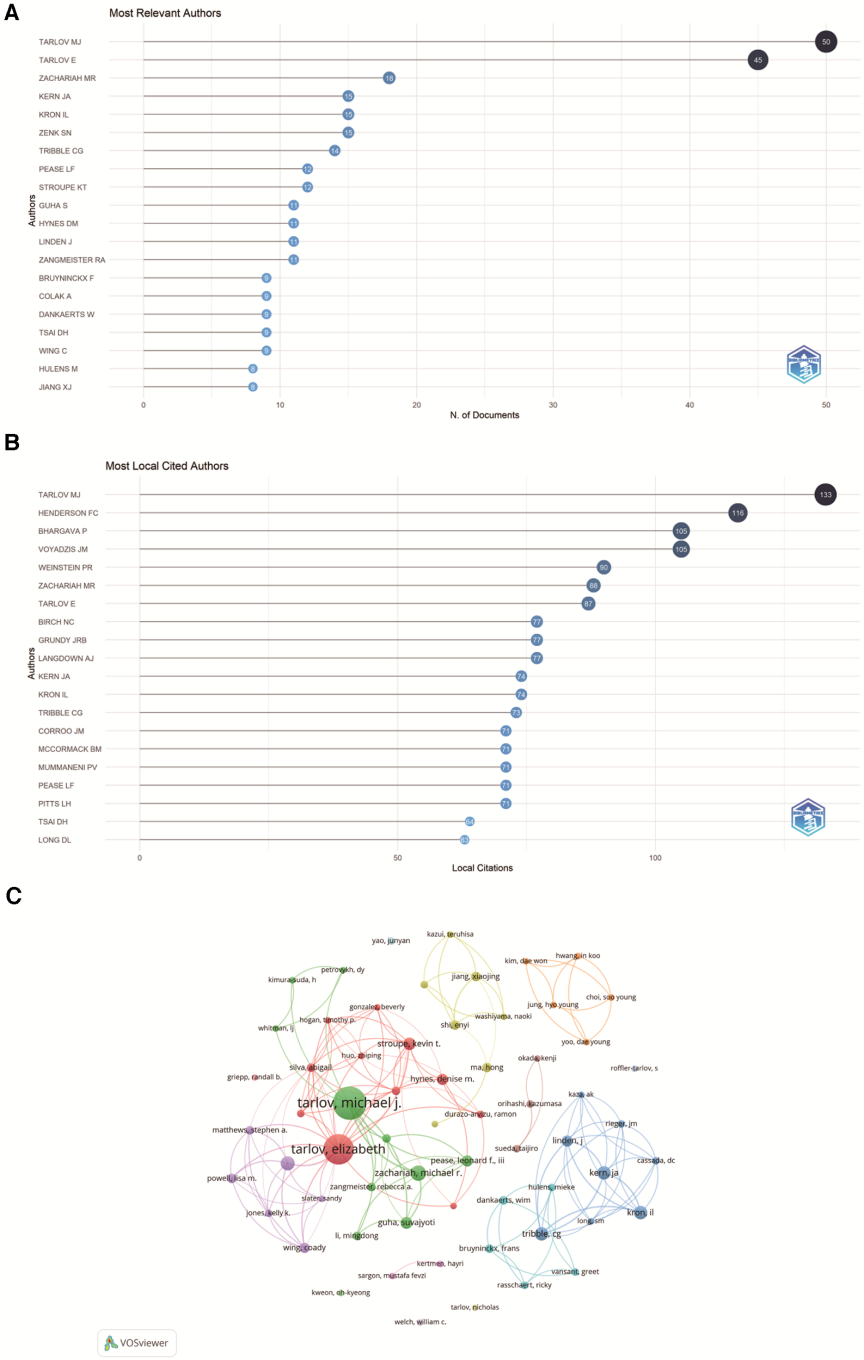
Authors analysis in STC research. (**A**) The top 20 Most Relevant Authors in STC research. (**B**) The top 20 Most Local Cited Authors in STC research. (**C**) Cooperation network map of authors in STC research.

Visual analysis of scholarly contributions and collaborations was achieved through the R-package “bibliometrix” and VOSviewer. Tarlov Mj and Tarlov E published the largest number of articles, signifying their broad recognition and authority in this field (Figure 7A). In terms of the most locally cited authors, Tarlov Mj ranked first (*n* = 133), followed by Henderson FC (*n* = 116) and Bhargava *P* (*n* = 105) (Figure 7B). The authors could be divided into seven clusters (Figure 7C). The first cluster encompassed Tarlov Mj, Zachariah Mr, Pease Lf and Tsai Dh, while the second cluster comprised Tarlov E, Stroupe Kt, Hynes Dm and Wing C. Overall, these two clusters formed the most significant cooperation network with each other.

### 2.8. Analysis of references and co-cited references

**Figure 8:**
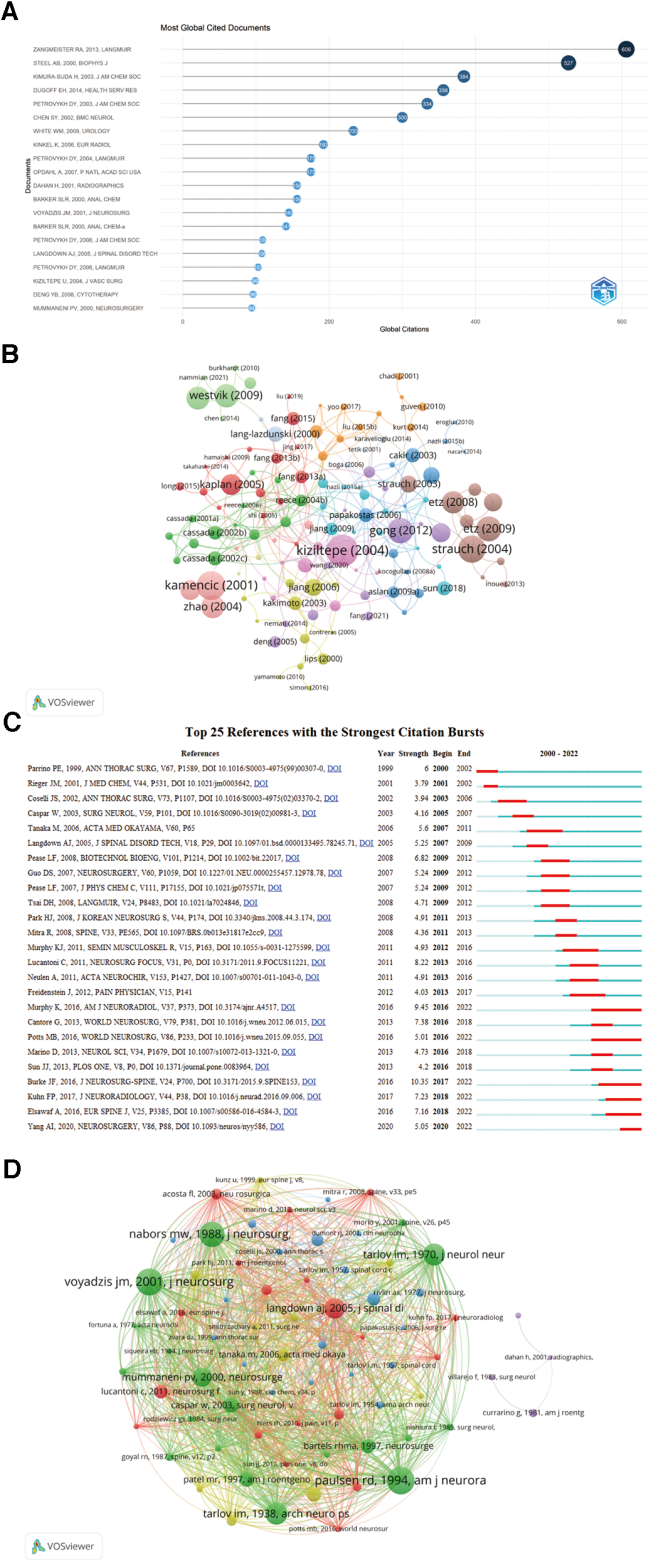
Analysis of documents and co-cited documents in STC research. (**A**) The top 20 most cited documents in STC research. (**B**) Cooperation network map of references in STC research. (**C**) The top 25 references with the strongest citation bursts in STC research. (**D**) Cooperation network map of co-cited references in STC research. STC, Sacral Tarlov cyst.

Zangmeister Ra's 2013 paper in *the Langmuir Journal* entitled “Characterization of polydopamine thin films deposited at short times by autoxidation of dopamine” was the most cited (total citations = 606), followed by Steel Ab's 2,000 article in *the Biophys Journal* entitled “Immobilization of nucleic acids at solid surfaces: effect of oligonucleotide length on layer assembly” (total citations = 527) and Kimura-Suda H's 2,003 article in the *Journal of the American Chemical Society* entitled “Base-dependent competitive adsorption of single-stranded DNA on gold” (total citations = 384) (Table 5, Figure 8A). Petrovykh Dy and Barker Slr also made significant contributions to this field, producing five of the top 20 highly cited references.

**Table 5:** The top 20 cited literature in STC.

Rank	Paper	Total citations	Total citations per year	DOI
1	Zangmeister Ra, 2013, Langmuir	606	60.6	10.1021/la400587j
2	Steel Ab, 2000, Biophys J	527	22.91	10.1016/S0006-3495 (00)76351-X
3	Kimura-Suda H, 2003, J Am Chem Soc	384	19.20	10.1021/ja035756n
4	Dugoff Eh, 2014, Health Serv Res	356	39.56	10.1111/1475-6773.12090
5	Petrovykh Dy, 2003, J Am Chem Soc	334	16.70	10.1021/ja029450c
6	Chen Sy, 2002, Bmc Neurol	300	14.29	10.1186/1471-2377-2-1
7	White Wm, 2009, Urology	233	16.64	10.1016/j.urology.2009.04.030
8	Kinkel K, 2006, Eur Radiol	192	11.29	10.1007/s00330-005-2882-y
9	Petrovykh Dy, 2004, Langmuir	175	9.21	10.1021/la034944o
10	Opdahl A, 2007, P Natl Acad Sci Usa	175	10.94	10.1073/pnas.0608568103
11	Dahan H, 2001, Radiographics	156	7.09	10.1148/radiographics.21.3.g01ma13575
12	Barker Slr, 2000, Anal Chem	156	6.78	10.1021/ac0008690
13	Voyadzis Jm, 2001, J Neurosurg	145	6.59	10.3171/spi.2001.95.1.0025
14	Barker Slr, 2000, Anal Chem-a	141	6.13	10.1021/ac000548o
15	Petrovykh Dy, 2006, J Am Chem Soc	109	6.41	10.1021/ja052443e
16	Langdown Aj, 2005, J Spinal Disord Tech	108	6.00	10.1097/01.bsd.0000133495.78245.71
17	Petrovykh Dy, 2006, Langmuir	103	6.06	10.1021/la050928a
18	Kiziltepe U, 2004, J Vasc Surg	99	4.95	10.1016/j.jvs.2004.03.032
19	Deng Yb, 2006, Cytotherapy	96	5.33	10.1080/14653240600760808
20	Mummaneni Pv, 2000, Neurosurgery	94	3.92	10.1097/00006123-200007000-00016

Visual analysis of highly cited articles was performed using the R-package “bibliometrix” and VOSviewer software. A total of 457 references with 10 or more citations were selected for the construction of the network map, revealing 128 coupled networks in this map. The different documents were represented by different points, while the point size represented the citation number of publications. As shown in Figure 8B, Kiziltepe (2004) ranked first, followed by Kamencic (2001) and Strauch (2004).

The CiteSpace 6.1.6 software was used to demonstrate the top 25 references with the strongest citation bursts. Figure 8C presents the burst strength of these documents, revealing a fluctuation rate between 3.79 and 10.35 and endurance strength from 1 to 6 years. “Murphy K, 2016, AM J NEURORADIOL, V37, P373” and “Potts MB, 2016, WORLD NEUROSURGERY, V86, P233” lasted the longest, approximately six years. The highest burst strength was “Burke JF, 2016, J Neurosurgery-Spine, V24, P700” (*n* = 10.35), followed by “Murphy K, 2016, Am J Neuroradiol, V37, P373” (*n* = 9.45) and “Lucantoni C, 2011, Neurosurgery Focus, V31, P0” (*n* = 8.22). A total of six co-citations had the most recent burst, which might be indicative of future STC research trends.

The visual analysis of co-cited references was performed using the VOSviewer software (Figure 8D). The clustering results of co-cited references in the field of STC research revealed six clusters (only references with citations ≥15 were included in the visualization) that were closely related to each other. Different documents were marked by different points, and the size of the point represented the link's strength and importance. The documents in the red cluster and green cluster were the most prominent in the network. In cluster 1 (red), Landdown aj (2005) had the most co-citations, followed by Lucantoni c (2011) and Acosta fl (2003). In cluster 2 (green), the three most significant documents were by Voyadzis jm (2001), Mummaneni pv (2000) and Caspar w (2003), displaying the greatest link strength.

### 2.9. Keywords and trend topics analysis

**Figure 9:**
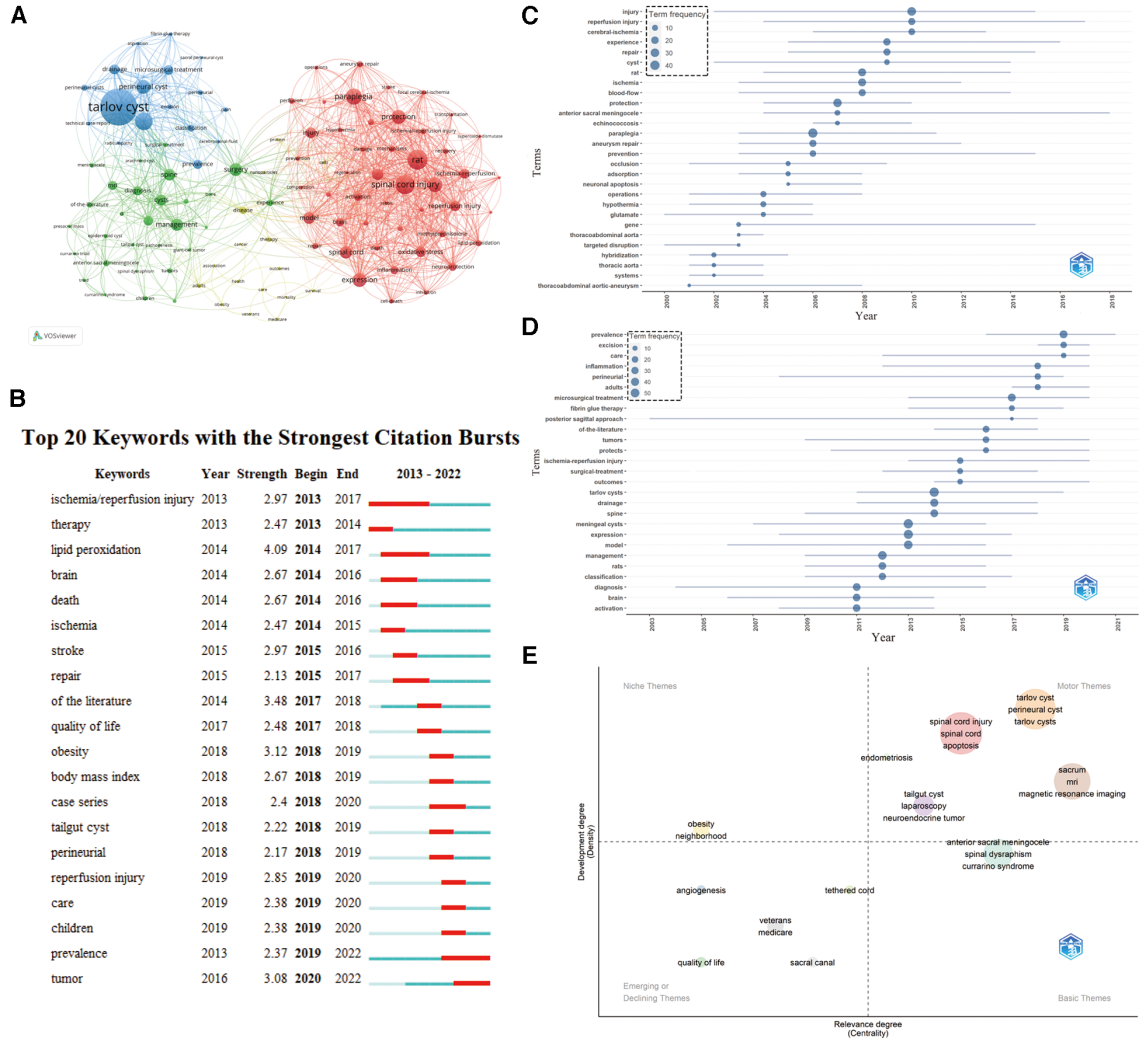
Keywords and trend topics analysis in STC research. (**A**) Cooperation network map of keywords in STC research. (**B**) The top 20 keywords with the strongest citation bursts in STC research. (**C**) and (**D**) Keyword trends from 2000 to 2022 in STC research. (**E**) Keyword topic map in STC research.

The top 20 high-frequency keywords were extracted from 930 publications. As shown in Table 6, the top five keywords were “Tarlov cyst”, “Rat”, “Spinal cord injury”, “Meningeal cyst” and “Paraplegia”. The co-occurrence map analysis of keywords was constructed using the VOSviewer software (Figure 9A), which unveiled current frontiers and future directions in STC research. The keyword frequency was limited to 10 or more, which yielded 134 keywords that were categorised into four clusters. The red cluster included keywords associated with pathogenesis and aetiology, such as “ischemia”, “inflammation”, “apoptosis”, “oxidative stress” and “spinal cord injury”. The green cluster included keywords related to current diagnostic methods and treatment options, such as “magnetic resonance imaging”, “diagnosis”, “management” and “surgery”. The blue cluster contained topics that primarily related to the nomenclature of diseases and surgical treatment, such as “Tarlov cyst”, “perineural cyst”, “meningeal cysts”, “classification”, “microsurgical treatment” and “drainage”. The yellow cluster represented prognosis and new technology prospects, including “outcomes”, “mortality”, “nanoparticles”, “cell” and “regeneration”.

**Table 6:** The top 20 keywords in STC.

Rank	Keywords	Frequency (*n*)	Rank	Keywords	Frequency (*n*)
1	Tarlov Cyst	135	11	Management	45
2	Rat	73	12	Spinal Cord	45
3	Spinal Cord Injury	70	13	Model	43
4	Meningeal Cyst	64	14	Spine	42
5	Paraplegia	59	15	Ischemia	41
6	Apoptosis	53	16	Injury	40
7	Expression	51	17	Cysts	39
8	Perineural Cyst	51	18	Oxidative Stress	38
9	Protection	51	19	Reperfusion Injury	38
10	Surgery	47	20	Drainage	36

The top 20 keywords with the strongest citation bursts were analysed using the CiteSpace software (Figure 9B). The burst strength of these keywords fluctuated between 2.13 and 4.09, and endurance strength was from 1 to 9 years. “Lipid peroxidation” had the strongest burst strength (*n* = 4.09), followed by “of the literature” (*n* = 3.48) and “obesity” (*n* = 3.12). “Ischemia/reperfusion injury” lasted approximately four years, ranking first. Furthermore, 12 keywords exhibited the strongest citation bursts in the past five years, wherein “prevalence” and “tumour” had the most recent burst. Figures 9C,D, rendered by the R-package “bibliometrix”, represent the keyword trends from 2000 to 2022. The size of the node represented the emergence degree of each keyword. R-package “bibliometrix” was also used to render a topic map (Figure 9E), wherein future trends and orientations could be embodied. The topic map was predicted by two axes, the horizontal axis for relevance degree (centrality) and the vertical axis for development degree (density). The map was divided into four quadrants, representing Niche Themes/Motor Themes/Emerging or Declining Themes/Basic Themes, respectively. The Niche Themes quadrant was highly popular, including keywords such as obesity and neighbourhood but had little relevance to the field. Motor Themes indicated that the research was closely related to the field and had a rapid development, including terms such as “Tarlov cyst”, “perineural cyst apoptosis”, “magnetic resonance imaging” and “spinal cord injury”. The Emerging or Declining Themes represented an emerging research direction or a study with declining attention, for example, “angiogenesis”, “quality of life” and “sacral canal”. Basic Themes represented valuable research directions that required further study, such as “anterior sacral meningocele”, “spinal dysraphism” and “currarino syndrome”.

## 3. Discussion

### 3.1. General information

Bibliometric analysis and its visualisation offer effective tools for assimilating information and comprehending the breadth of relevant research within a field (26). In this study, a bibliometric analysis of 930 articles on STCs from the WOSCC database (2000–2022) was conducted to evaluate the current research landscape, identify research hot spots and anticipate future trends.

Despite the relatively limited literature, the publication volume of STC has been on the rise in recent years. The increasing publication volume may reflect a growing interest in various aspects of STC, including clinical management, pathophysiology, and treatment strategies. The substantial attention and citations indicate that researchers recognize the achievements in the STC field and anticipate further progress. Analysis of the number of publications by country reveals that the USA has the highest number of publications, followed by China and Turkey, a trend that is mirrored in the total number of citations. Thus, the literature published by these countries needs to be focused on when studying this field. In terms of national collaborations, countries such as the USA and Japan have strong collaborations with other countries, whereas China and Turkey, despite their high number of publications, have weak international collaborations. Moreover, the published literature from China and Turkey is mainly based on domestic collaborations. Hence, there is still a need for enhanced international engagement for advancing STC research. The institutional analysis revealed that the University of Illinois, the University of Maryland and the National Institute of Standards & Technology in the USA are the highest contributors to this field, reflecting their substantial research depth. In terms of journal research, STC-related studies were mainly published in specialised journals such as *World Neurosurgery*, *Journal of Neurosurgery-Spine*, *Journal of Vascular Surgery* and *European Spine Journal*. Notably, the *Journal of Neurosurgery-Spine* boasts the highest citation count, indicating its recognition and quality within the field. Although the volume of literature published in *Spine* is not high, it ranks second in citations. Furthermore, The volume of publications by authors and the distribution of authorship reveal influential research groups and potential collaborative relationships. Tarlov Mj had the highest number of publications, citations and co-citations, identifying him as an influential and prolific figure in this field. Additionally, Tarlov E, Zachariah Mr, Kern Ja and others have also made important contributions to the advancement of this field.

### 3.2. Research hotspots and trends

According to the keyword co-occurrence map, four categories of keywords, namely disease diagnosis, mechanism exploration, disease definition and treatment methods, were elucidated. To a certain extent, these keywords also represent the research hotspots in the field. For instance, the frequency and clustering of keywords can reflect the research hotspots of a field, whereas the keyword burst analysis and trend analysis can reflect the research trends and possible future directions of a field (27). Our multifaceted analysis of keywords revealed that “Tarlov Cyst”, “Spinal Cord Injury”, “Meningeal Cyst”, and “Paraplegia” were among the more frequently used keywords. Additionally, the thematic analysis revealed that “Tarlov cyst”, “perineural cyst apoptosis”, “magnetic resonance imaging”, “spinal cord injury”, “anterior sacral meningocele”, “spinal dysraphism” and “currarino syndrome” as important areas of research. The keyword trend graph shows that before 2010, the hotspots of research were mainly related to the definition and treatment of STC, such as “injury”, “repair”, “cyst”, “operation”, “paraplegia”, and others. After 2010, STC research focused on mechanism studies, diagnosis, and clinical observation, such as “inflammation”, “diagnosis”, “exercise” and “prevalence”. Nevertheless, the definition and treatment of diseases remain the focus of attention. The analysis of keyword bursts also shows the trends and directions of research. In the early days of STC research, studies focused primarily on the pathogenesis and treatment of the disease, using keywords such as “ischemia/reperfusion injury”, “therapy”, “lipid peroxidation”, and “repair”. In the last five years, the pathogenesis and treatment of STC and its association with tumours are increasingly being studied, involving terms such as “perineurial”, “reperfusion injury” and “prevalence”. Collectively, the current research hotspots in STC are focused on the definition, diagnosis, pathogenesis and treatment of the disease, providing clear directions for future focus.

#### 3.2.1. Definition

The definition of STC constitutes a foundational aspect of early research. It is generally accepted that a Tarlov cyst is a perineural cyst and because it occurs mainly in the lumbosacral region, the sacral cyst also becomes an important component of Tarlov cysts. While studies suggest a high prevalence of Tarlov cysts (approximately 1.5% of the general population), symptoms are observed in only 13% of cases (28). A meta-analysis reported a global prevalence of 4.27%, with a relatively narrow confidence interval (CI) (95% CI: 2.56–6.38). Similarly, Larsen et al. (29) suggested a maximum general prevalence of 17.65%. Currently, STC is defined as a perineural cyst between the perineurium and endoneurium of the sacral nerve, originating in the vicinity of the dorsal root ganglion (DRG), often at the S2 site (29). Symptomatic STC mainly manifests as nerve root pain, low back pain, abnormal sensation, abnormal urine or bowel function, leg weakness and sexual dysfunction (11, 30). A clear understanding of STC's definition, epidemiology and potential symptoms is crucial for mechanistic research and later-stage treatments.

#### 3.2.2. Pathogenesis

The pathogenesis of STC remains unclear, which is the main reason for the continued research into its mechanism. While various perspectives have been proposed by experts, the “ball-valve” theory emerges as a reasonable explanation for the development of STC and is accepted by scholars. This mechanism suggests that cerebrospinal fluid flows through the unidirectional valve into the cavity between the nerve bundle membrane and the endoneurium and accumulates as a cyst, thereby promoting the development and progression of STC (31). The herniated area often forms a one-way valve-like structure, trapping cerebrospinal fluid within the cyst and preventing outflow, causing the cyst to gradually expand and elicit symptoms (32). The symptoms of STC are correlated to its location, size and association with nerve roots (33). Essentially, Tarlov cysts manifest as dilated nerve roots due to pathologically increased hydrostatic pressure (HP) in the spinal canal (34). Nevertheless, the mechanisms underlying STC necessitate further exploration for a comprehensive understanding.

#### 3.2.3. Diagnosis

Similarly, the diagnosis of STC remains ambiguous. Often, asymptomatic STC cases are found incidentally through magnetic resonance imaging (MRI) of the spine (28). MRI clearly shows the location, shape, size and number of cysts, and can also identify intra-sacral tumours. Importantly, STC is a frequently overlooked disease in clinical practice, requiring differentiation from lumbar disc herniation, sacral canal tumours and lumbar spinal stenosis. Studies reveal that STC is underdiagnosed for various reasons, mainly due to persistent clinician misconceptions and biases (8). A questionnaire-based study revealed the main symptoms of STC, such as perineal symptoms, bowel symptoms, bladder symptoms and anogenital sphincter problems, which are exacerbated by sedentary behaviour, walking and exertion. Moreover, in severe cases, the individual has to cease work and social activity (35). Nevertheless, it is important to differentiate it from, for example, low back pain and sciatica. Thus, attention needs to be paid to not only imaging results but also to the patient's symptoms, signs and necessary laboratory tests to ensure efficient differential diagnosis. An accurate diagnosis is imperative to develop an effective treatment strategy.

#### 3.2.4. Treatment

A myriad of treatment options for STC exist, yet controversies persist, and a unified understanding remains elusive. Treatment options include fibrin glue injection, cyst drainage, open surgery and minimally invasive surgery. A study from China compared the clinical outcomes of microscopic cyst openings with stacked tiles, C-arm fluoroscopy-guided percutaneous fibrin gel injection and conservative treatment of STC through retrospective analysis. The results revealed that C-arm fluoroscopy-guided percutaneous fibrin gel injection therapy had better outcomes, highlighting its recommendation potential (36). Furthermore, Huang et al. (37) retrospectively studied the efficacy of subcutaneous infusion port surgery in five patients with STC and found significant pain relief in all patients with no complications and adverse effects. Cyst repair or cerebrospinal fluid tamponade has also been reported to be sufficient to relieve symptoms in patients with STC (38). It is worth noting that our review of the literature and bibliometric analysis has shown that, in recent years, there has been a surge in surgical approaches for the treatment of STC, with increasing success and less invasiveness. However, standardised criteria for STC surgery indications are lacking. Clinical symptoms, such as pain, affecting normal life, bowel and urinary dysfunction, and the absence of conditions like lumbar disc herniation and spinal tumours should warrant surgical intervention. For complications of Tarlov cysts (STC), such as intracystic hemorrhage, surgery is considered an effective treatment method (39). Additionally, for cysts with a diameter exceeding 1.5 centimeters, surgical intervention is also recommended. A systematic review published in 2018 summarized the current evidence on surgical treatment of STC and suggested that surgery for symptomatic STC could be an effective option for partial or complete symptom relief. However, this was not the case for larger cysts (40). The latest meta-analysis found that patients who underwent surgical intervention had a postoperative complication rate of 16.9% (11.8–22.7) and a cyst recurrence rate of 8.5% (3.5–15.4). The most common complications were the occurrence of surgical site infection and/or cerebrospinal fluid leakage (41). Currently, surgery is the primary effective treatment for symptomatic STC.

With the advent of minimally invasive concepts, the more mainstream surgical procedures for STC are now mainly microscopic and endoscopic minimally invasive procedures. In a retrospective study, Yucesoy et al. (42) found that cyst microdissection significantly improved the symptoms of STC. Moreover, it was easy to perform and safe, with no major complications observed. Additionally, a new microsurgical sealed procedure for STC has shown high clinical efficacy. After a mean follow-up period of 44.69 months in 265 patients, 94.14% of the cysts had shrunk or disappeared, with an excellent rate of 80.73% of patients three years after discharge (43). These results demonstrate the importance of microsurgical techniques in the management of STC. Huang et al. (44) studied microscopic methods of cyst separation and orifice closure in 35 patients, wherein 33 cases of complete or substantial resolution of symptoms were observed. It is, therefore, evident that microsurgical techniques are an important approach and that innovation and improvement in this avenue is a promising approach to the treatment of STC. Another widely accepted minimally invasive technique is the endoscopically operated approach. Endoscopy technology has made rapid progress, and its applications cover degenerative diseases, trauma, inflammation, and tumors. This technique has the advantages of less trauma, faster recovery and fewer complications (45–48). In recent years, some scholars have tried to apply endoscopic technique to the treatment of STC. For instance, Zhang et al. (49) used percutaneous endoscopic surgery to safely and effectively resect STC, and this novel minimally invasive strategy may hold great promise for symptomatic STC. However, this evidence stems from a case report, warranting validation through larger trials at later stages. Kang et al. (50) also reported a case wherein STC was treated using a percutaneous double portal endoscopic windowing, and the patient showed significant improvement in motor weakness and radiating pain after decompression. Wang et al. (51) retrospectively analysed 15 patients with STC treated with spinal endoscopy from August 2018 to January 2020 and found that 85.7% of patients had significant pain relief after the procedure, and postoperative MRI showed satisfactory cyst filling with no recurrence of cysts. Thus, spinal endoscopic techniques demonstrate preliminary clinical efficacy, offering enhanced patient safety and minimal invasiveness, making them a worthwhile avenue for promotion. We believe that the science and rationality of endoscopy technology can be discussed from the perspective of fluid mechanics, so as to provide guidance for the study of endoscopic surgery mechanism (52), this is what we will study in depth next.

## 4. Limitations

As far as we know, this study represents the first comprehensive bibliometric analysis in the field of STC. Nevertheless, there are still some potential limitations in our research. Firstly, the dynamic nature of databases implies that conclusions drawn from data obtained at different time points may vary. Our analysis, in this case, only covers literature from 2000 to 2022. Secondly, we focused exclusively on the WOSCC database; however, this is not expected to significantly impact the overall direction of the study. Additionally, in our author analysis, we made efforts to attribute authorship to similar authors. However, we cannot definitively determine whether several authors with the same name, automatically merged by the software, belong to the same researcher.

## 5. Conclusions

Despite modest annual publication rates, the STC field sustains a consistent and increasing publication trend, indicating that it is a disease that cannot be ignored. For the past 20 years, USA has led in publications and citations, followed by China and Turkey. Furthermore, the University of Illinois is the most published institution, *World Neurosurgery* is the most published journal and Tarlov Mj is the most published, cited and co-cited author, all important contributors to this field. Moreover, enhanced cooperation between countries, institutions and authors is needed to promote the development of this field. Furthermore, in recent years, research hotspots in the field of STC have focused on the definition, pathogenesis, diagnosis and treatment of the disease, with surgical treatment being the most important approach. Different surgical methods are currently available, and microscopic and endoscopic techniques are poised to shape the future of STC treatment. As research in this field advances, it will continue to focus on the aspect of clinical efficacy.

## Data Availability

The original contributions presented in the study are included in the article/Supplementary Material, further inquiries can be directed to the corresponding authors.
